# Cost-effectiveness analysis of eight first-line treatments for metastatic hormone-sensitive prostate cancer in China

**DOI:** 10.3389/fphar.2025.1684966

**Published:** 2025-11-28

**Authors:** Zhou Han, Youli Xi, Jian Hu, Ye Wang, Huanyu Ni

**Affiliations:** 1 Department of Pharmacy, Nanjing Drum Tower Hospital, The Affiliated Hospital of Nanjing University Medical School, Nanjing, Jiangsu, China; 2 Nanjing Medical Center for Clinical Pharmacy, Nanjing, Jiangsu, China; 3 Pharmacy Department, The Second Affiliated Hospital of Nanjing Medical University, Nanjing, Jiangsu, China; 4 Sir Run Run Shaw Hospital, School of Medicine, Zhejiang University, Hangzhou, Zhejiang, China

**Keywords:** abiraterone, ADT, cost-effectiveness analysis, mHSPC, partitioned survival model

## Abstract

**Background:**

The treatment of metastatic hormone-sensitive prostate cancer (mHSPC) has shifted from androgen deprivation therapy (ADT) alone to doublet or triplet regimens building on ADT. However, the cost-effectiveness analysis of first-line treatments for mHSPC in China is uncertain. This study aims to perform a 10-year horizon health economic evaluation to comparatively analyze the cost-effectiveness of eight treatment regimens for mHSPC from the perspective of China’s healthcare system, including (1) ADT alone and ADT plus one of the following: (2) docetaxel, (3) abiraterone, (4) apalutamide, (5) enzalutamide, (6) rezvilutamide, (7) darolutamide and docetaxel, (8) abiraterone and docetaxel.

**Methods:**

Partitioned survival model was developed to evaluate the cost-effectiveness of eight first-line treatment regimens for mHSPC. Drug costs were primarily extracted from pharmaceutical databases. The key outcomes were quality adjusted life years (QALYs), costs and the incremental cost-effectiveness ratio (ICER). Willingness-to-pay (WTP) threshold was set as three-time China’s gross domestic product (GDP) *per capita* (US$38,024) per QALY.

**Results:**

For costs, the 10-year cost estimates ranged from US$120,844 for ADT alone to US$216,294 for darolutamide plus ADT with docetaxel. For clinical effectiveness, enzalutamide plus ADT yielded the highest QALYs (4.55), while ADT alone gained lowest QALYs (3.01). For cost-effectiveness, the three treatment regimens of ADT alone, abiraterone plus ADT and enzalutamide plus ADT constituted the cost-effectiveness frontier. Abiraterone plus ADT emerged as the most cost-effective strategy, indicative of an ICER of US$17437.16 per QALY, substantially below WTP threshold.

**Conclusion:**

Abiraterone plus ADT was likely to be cost-effective for mHSPC treatment at a WTP threshold of three-time *per capita* GDP per QALY.

## 1 Introduction

Prostate cancer is a public health challenge in China, characterized by a rapidly increasing incidence, a lower survival rate (Han et al., 2024), and a substantial burden of disability-adjusted life years (DALYs) (Sun et al., 2020). A distinctive feature of the disease in China is the high proportion of patients presenting with metastatic hormone sensitive prostate cancer (mHSPC) at initial diagnosis—up to 68% (Wang et al., 2025), in stark contrast to approximately 16% in the United States. With multiple treatment regimens available for mHSPC in clinical practice, conducting cost-effectiveness analyses (CEA) tailored to the Chinese population is critically important for informing healthcare decisions.

mHSPC constitutes a pivotal therapeutic window in the prostate cancer continuum. During this stage, while metastases are present, tumor cells retain sensitivity to androgen deprivation therapy (ADT), creating a critical therapeutic window for clinical intervention (Hussain et al., 2024). Evidence-based studies confirm that early intensification of systemic therapy at the mHSPC stage significantly delays disease progression, prolongs overall survival (OS), and improves quality of life. Compared to the castration-resistant prostate cancer (CRPC) phase, interventions during this window confer substantially greater survival benefits (Burdett et al., 2019; Hussain et al., 2023; Hussain et al., 2024). Consequently, optimizing mHSPC treatment regimens constitutes not only a core challenge in clinical decision-making but also a critical juncture for improving overall disease trajectory in metastatic prostate cancer.

Over the past two decades, ADT monotherapy has been established as the cornerstone initial treatment for mHSPC (Bolla et al., 1997). However, its efficacy remains limited, with most patients progressing to metastatic castration-resistant prostate cancer (mCRPC) within a median timeframe of 1–2 years (Hellerstedt and Pienta, 2002; Katzenwadel and Wolf, 2015). Very recent years have witnessed a paradigm shift in the management of mHSPC. Numerous phase III trials, including LATITUDE, ENZAMET, CHAARTED, ARCHES and PEACE-1 (Fizazi et al., 2017; Armstrong et al., 2019; Davis et al., 2019; Fizazi et al., 2019; Chi et al., 2021; Armstrong et al., 2022; Fizazi et al., 2022; Gu et al., 2022; Smith et al., 2022), have investigated dual or triple therapy regimens based on ADT. These studies conclusively demonstrate that combining ADT with docetaxel, androgen receptor pathway inhibitors (ARPIs), or ARPIs plus docetaxel significantly prolongs OS, enhances survival outcomes, and maintains favorable tolerability profiles.

However, the rapid evolution of diverse combination therapies for mHSPC treatment, while improving patient survival outcomes, presents new challenges for clinical decision-making. The absence of head-to-head comparative trials among these regimens perpetuates uncertainty regarding the optimal treatment strategy for mHSPC. Moreover, substantial variations in therapeutic approaches inevitably lead to divergent cost implications, further escalating burdens on healthcare financing systems. Previous cost-effectiveness analysis of first-line mHSPC therapies did not incorporate triplet regimens (Sung et al., 2021; Wang L. et al., 2022). More importantly, economic evaluations of these strategies within China’s healthcare context remain particularly scarce. To address these gaps, we perform a cost-effectiveness analysis of eight first-line mHSPC treatments to confirm the optimal therapeutic regimen in China using a partitioned survival modeling approach.

## 2 Materials and methods

### 2.1 Model overview

**FIGURE 1:**
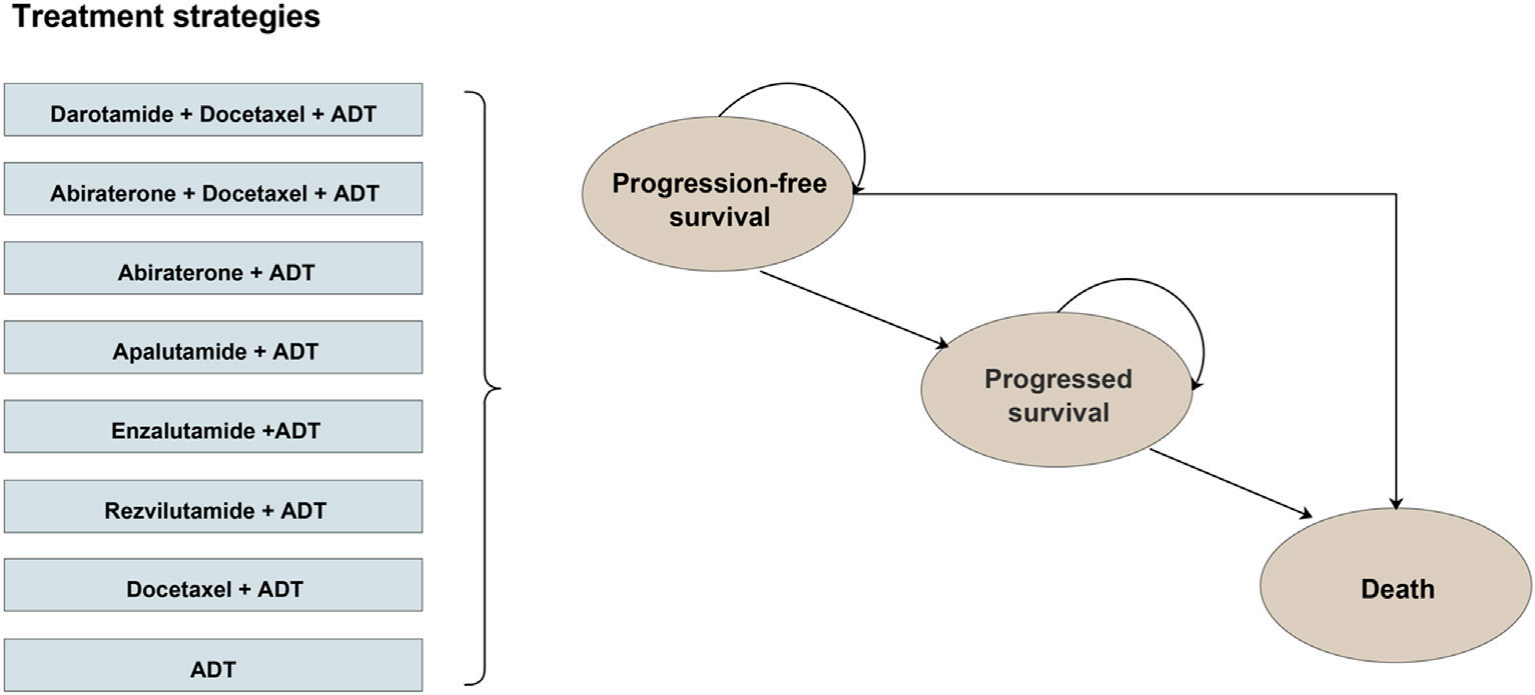
Model structure diagram of eight first-line treatments for metastatic hormone-sensitive prostate cancer (mHSPC).

A partitioned survival model was selected over a semi-Markov model for this analysis. This choice was driven by the nature of the available data, as the model was developed on reconstructed individual patient data (IPD) from published Kaplan-Meier (KM) curves rather than original IPD. Transition probabilities between health states comes from long-term clinical outcomes (Woods et al., 2020). The model included 3 health states: progression-free, progression to mCRPC and death (Figure 1). Once in progression, patients could remain in that state or transition to death (Supplementary Table S1). Given the ≤10-year median survival in mHSPC populations, this study was designed for a time horizon of 10 years with a cycle of 4 weeks (Wang L. et al., 2022). The model adopted in this study considered a hypothetical cohort of 60-year-old men with newly diagnosed mHSPC and one of the following treatment regimens was adopted: (1) ADT alone; (2) docetaxel plus ADT; (3) abiraterone plus ADT; (4) abiraterone plus ADT with docetaxel; (5) apalutamide plus ADT; (6) enzalutamide plus ADT; (7) rezvilutamide plus ADT; or (8) darolutamide plus ADT with docetaxel (Supplementary Table S2). For each treatment regime, the model produces different disease outcomes, thus affecting costs and quality adjusted life years (QALYs). The two primary model outputs Costs and QALYs of each treatment regimen were estimated over the 10 years horizon, discounted at 5% per year and used to calculate the incremental cost-effectiveness ratio (ICER) (Guan et al., 2022). Costs were obtained from the China health payer perspective. One to three times the annual gross domestic product (GDP) *per capita* was used as the lower and higher boundaries of the willingness-to-pay (WTP) threshold in China (Su et al., 2020; Liu et al., 2020). This model was programmed in Tree-Age Pro 2023 (Tree-Age Software, Williamstown, MA, United States), and the methodological rigorousness of studies was assessed with the Consolidated Health Economic Evaluation Reporting Standards (Husereau et al., 2022) (CHEERS) (Supplementary Table S3).

### 2.2 Progression of metastatic disease

Engauge Digitizer software was used to extract the data of survival rate from published KM curves for OS and progression-free survival (PFS) by converting graphical plots into numerical values. Based on the number at risk and the total number of events, we then accurately reconstructed IPD. The IPD was validated by comparing the hazard ratios or median survival times calculated from the reconstructed data with those reported in the original publications. If data on radiographic PFS were not available, data on clinical PFS were used in this study. The virtual patient-level of OS and PFS data were reconstructed using the standard statistical methodology described by Guyot (Guyot et al., 2012).

**FIGURE 2:**
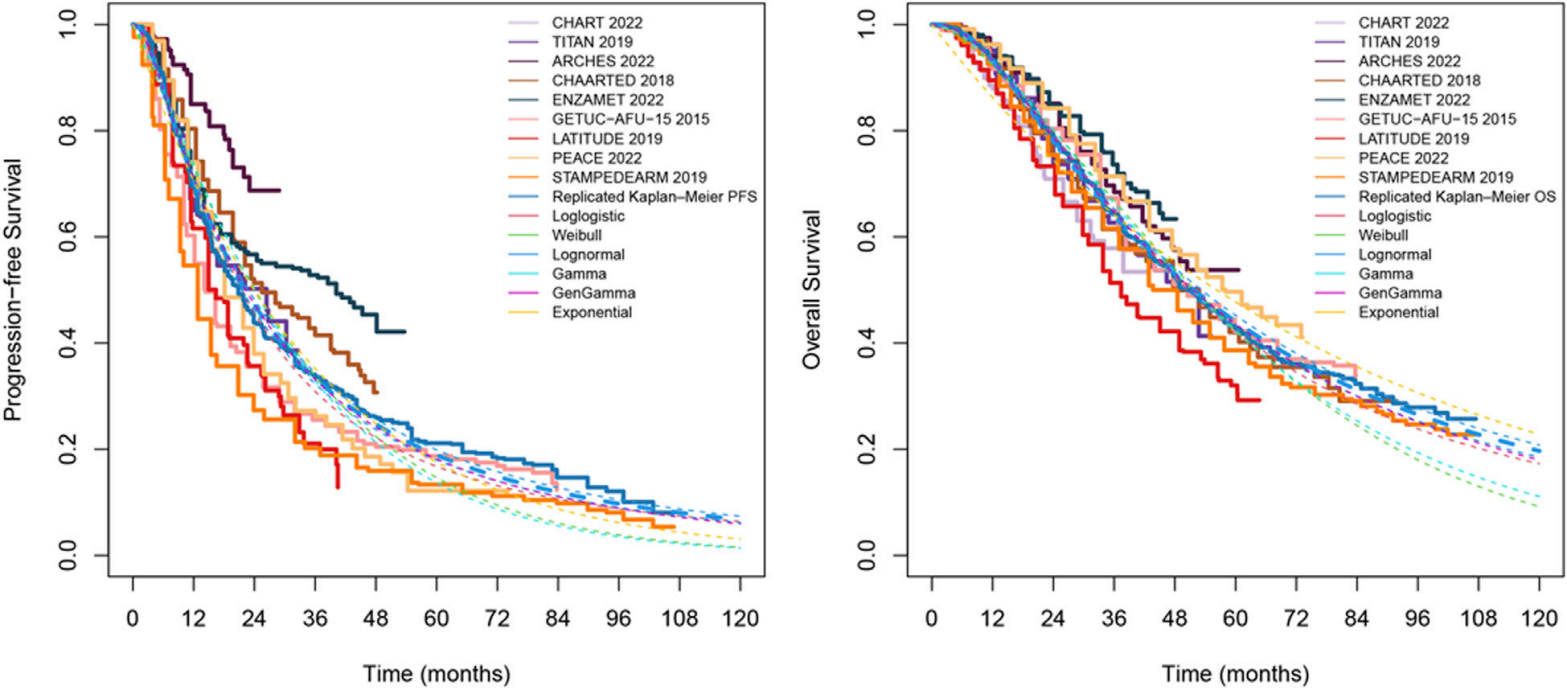
Kaplan-Meier curves for PFS and OS under ADT alone treatment were replicated by pooling data from nine clinical trials: GETUG-AFU, STAMPEDE, ENZAMET, LATITUDE, ARCHES, TITAN, PEACE-1, CHAARTED, and ARASENS. The solid, dashed, and dotted lines represent the mean estimates, upper bounds, and lower bounds of the 95% confidence intervals, respectively.

ADT monotherapy is the most commonly used control regimen in randomized controlled trials (RCTs) for mHSPC. Thus, in this study the ADT monotherapy group was set as reference treatment (Figure 2; Supplementary Figure S1). To evaluate the health outcomes, this study used multiple standard parametric survival models, including exponential, Weibull, Gompertz, log-logistic, and log-normal distributions. On the basis of visual fit and statistical goodness-of-fit (Akaike Information Criterion and Bayesian Information Criterion), Log-logistic model and Log-normal model were chosen to fit survival curves of different treatment regimens. The constant transition probability from CRPC to death for all treatment regimens was assumed and estimated by calibrating to the OS curves. The China life tables were used to estimate the risk of all-cause mortality (Supplementary Table S4).

### 2.3 Treatment strategies

Docetaxel was administered at 75 mg/m^2^ every 3 weeks for six cycles. Abiraterone was administered at a dose of 1,000 mg daily, enzalutamide at 160 mg daily, and apalutamide at 240 mg daily, darolutamide at 600 mg twice daily, rezvilutamide at 240 mg daily until progression. For all treatment regimens, ADT (goserelin, leuprolide or triptorelin) was administered until progression. We included grade 3 or above adverse events and assumed that adherence to treatment was 100%.

### 2.4 Medical costs and health utilities

Medical Costs: This model only included the direct medical costs, e.g., costs of drugs, costs of management, costs of adverse reaction treatment, follow-up costs, terminal care costs, costs of best supportive care. All medical expenses are denominated in US dollars (1 USD = 7.12 CNY) and adjusted to 2024 values based on China’s Medical Consumer Price Index. The model inputs of clinical data, cost and utility estimates are summarized in Tables 1, 2, and detailed information are as follows. The prices of drugs were obtained from Yaozh and Menet (Yaozh, 2024; Menet, 2024). The specific prices of drugs for mHSPC treatment are provided in Supplementary Table S5. To calculate the docetaxel dose, the average body surface area for Chinese patients was assumed to be 1.72 m^2^. The proportion of receiving specific subsequent treatment after disease progression to CRPC varies significantly across eight treatment regimens for mHSPC, which are obtained from the corresponding RCTs (Supplementary Table S6). For the darolutamide plus ADT with docetaxel regimen, 56.8% of patients who progressed to CRPC received specific subsequent therapy: abiraterone plus ADT (35.6%), enzalutamide plus ADT (15.2%), docetaxel plus ADT (32.7%) (Smith et al., 2022; Fizazi et al., 2019; Davis et al., 2019). The others only received best supportive care. Follow-up costs primarily encompassed laboratory surveillance expenditures, including prostate-specific antigen (PSA) panel analyses, serum testosterone assays, complete blood counts (CBC), and comprehensive metabolic panels, alongside protocol-driven advanced imaging modalities. During the initial 6-month therapeutic phase, patients underwent quarterly imaging assessments comprising bone scintigraphy, computed tomography (CT), and magnetic resonance imaging (MRI). Subsequent monitoring transitioned to semi-annual imaging cycles utilizing these same modalities. The details are listed in Supplementary Table S7. Adverse reaction management costs were estimated for common AEs experienced by mHSPC patients during pharmacotherapy. These include fatigue, asthenia, back pain, neutropenia, bone pain, hypertension, hypokalemia, elevated ALT, elevated AST, and febrile neutropenia. These adverse events require corresponding therapeutic interventions and management, which may impact patients’ healthcare costs (Supplementary Table S8).

**TABLE 1:** Summary of main clinical inputs.

Parameter	Values
Clinical inputs	
Survival model of Darotamide + Docetaxel + ADT	
Log-logistic model of PFS	shape, 1.7795 (se, 0.1032); scale, 66.0907 (se, 3.4510); AIC, 2685
Log-normal model of OS	meanlog, 4.2325 (se, 0.0586); sdlog, 0.9993 (se, 0.0525); AIC, 2672
Survival model of Abiraterone + Docetaxel + ADT	
Log-logistic model of PFS	shape, 1.5252 (se, 0.1049); scale, 40.1552 (se, 3.0242); AIC, 1538
Log-logistic model of OS	shape, 1.8392 (se, 2.1602); scale, 65.6037 (se, 4.9644); AIC, 1254
Survival model of Abiraterone + ADT	
Log-logistic model of PFS	shape, 1.3868 (se, 0.0343); scale, 40.5162 (se, 2.1041); AIC, 12,314
Log-logistic model of OS	shape, 1.6581 (se, 0.0431); scale, 60.9455 (se, 2.5857); AIC, 12,731
Survival model of Apalutamide + ADT	
Log-normal model of PFS	meanlog, 3.1806 (se, 0.0818); sdlog, 1.1943 (se, 0.0709); AIC, 1605
Log-normal model of OS	meanlog, 4.2623 (se, 0.0751); sdlog, 1.0293 (se, 0.0655); AIC, 1852
Survival model of Enzalutamide + ADT	
Log-normal model of PFS	meanlog, 4.0384 (se, 0.0631); sdlog, 1.2591 (se, 0.0370); AIC, 8108
Log-logistic model of OS	shape, 1.7852 (se, 0.0718); scale, 79.0892 (se, 4.5142); AIC, 5,923
Survival model of Rezvilutamide + ADT	
Log-normal model of PFS	meanlog, 3.9209 (se, 0.1393); sdlog, 1.2390 (se, 0.1139); AIC, 815
Log-normal model of OS	meanlog, 4.2676 (se, 0.1438); sdlog, 1.1648 (se, 0.1146); AIC, 785
Survival model of Docetaxel + ADT	
Log-logistic model of PFS	shape, 1.6660 (se, 0.0284); scale, 21.1201 (se, 0.7501); AIC, 20,670
Log-logistic model of OS	shape, 1.7819 (se, 0.0311); scale, 53.7366 (se, 1.6337); AIC, 25,352
Survival model of ADT	
Log-logistic model of PFS	shape, 1.5624 (se, 0.0189); scale, 28.5109 (se, 1.0186); AIC, 41,639
Log-normal model of OS	meanlog, 3.9543 (se, 0.0315); sdlog, 0.9846 (se, 0.0123); AIC, 38,887

**TABLE 2:** Summary of main medical costs, utility values, and other parameters.

Parameter	Base case	Range		Distribution	References
		Low	High		
Drug cost (US$)					
Abiraterone/table	1.500	0.730	15.230	Gamma	Yaozh (2024); Menet (2024)
Enzalutamide/table	6.770	5.416	8.124	Gamma	
Docetaxel/mg	2.120	1.696	2.544	Gamma	
Apalutamide/table	6.800	5.440	8.160	Gamma	
Darotamide/table	6.920	5.536	8.304	Gamma	
Rezvilutamide/table	9.900	7.920	11.880	Gamma	
Prednisone/mg	0.010	0.008	0.012	Gamma	
Goserelin/mg	33.380	26.704	40.056	Gamma	
Leuprorelin/mg	33.850	27.080	40.620	Gamma	
Triptorelin/mg	27.020	21.616	32.424	Gamma	
Cost of management/cycle (US$)	345.000	241.500	448.500	Gamma	Assumpted
Cost of follow up/cycle (US$)	1415.264	990.685	1839.843	Gamma	Assumpted
Cost of terminal care (US$)	12,721	8904.7	16,537.30	Gamma	Wang Y. et al. (2022)
Cost of best supportive care/cycle	353.00	247.10	458.90	Gamma	Wang Y. et al. (2022)
Body surface area	1.720	1.500	1.900	Gamma	Zhang et al. (2024)
Cost of managing adverse events	1383	1244.7	1521.30	Gamma	Assumpted
Subsequent treatment proportion					
Darotamide + Docetaxel + ADT	0.568	0.511	0.625	Beta	Fizazi et al. (2017); Armstrong et al. (2019); Davis et al. (2019); Fizazi et al. (2019); Chi et al., (2021); Armstrong et al., (2022); Fizazi et al. (2022); Gu et al. (2022); Smith et al. (2022)
Abiraterone + Docetaxel + ADT	0.460	0.414	0.506	Beta	
Abiraterone + ADT	0.300	0.270	0.330	Beta	
Apalutamide + ADT	0.470	0.423	0.517	Beta	
Enzalutamide + ADT	0.670	0.603	0.737	Beta	
Rezvilutamide + ADT	0.270	0.243	0.297	Beta	
Docetaxel + ADT	0.700	0.630	0.770	Beta	
ADT	0.700	0.630	0.770	Beta	
Risk of adverse events (grade III–IV)					
Darotamide + Docetaxel + ADT	0.700	0.630	0.770	Beta	
Abiraterone + Docetaxel + ADT	0.630	0.567	0.693	Beta	
Abiraterone + ADT	0.480	0.432	0.528	Beta	
Apalutamide + ADT	0.530	0.477	0.583	Beta	
Enzalutamide + ADT	0.440	0.396	0.484	Beta	
Rezvilutamide + ADT	0.510	0.459	0.561	Beta	
Docetaxel + ADT	0.680	0.612	0.748	Beta	
ADT	0.430	0.387	0.473	Beta	
Health utility					
Utility of PFS	0.865	0.779	0.952	Beta	Sathianathen et al. (2019)
Utility of PFS with docetaxel	0.800	0.720	0.880	Beta	
Utility of PFS with ADT	0.830	0.747	0.913	Beta	
Utility of progressed patients	0.450	0.405	0.495	Beta	
Grade Ⅲ/Ⅳ anemia	0.119	0.107	0.131	Beta	
Grade Ⅲ/Ⅳ fatigue	0.473	0.426	0.520	Beta	
Grade Ⅲ/Ⅳ backpain	0.063	0.057	0.069	Beta	
Grade Ⅲ/Ⅳ neutropenia	0.131	0.118	0.144	Beta	
Grade Ⅲ/Ⅳ bone pain	0.067	0.060	0.074	Beta	
Discount rate	0.05	0.00	0.08	Fixed in PSA	Liu et al. (2020)

Health Utilities: According to the corresponding literature, the utility values for the PFS and progressive disease (PD) state were 0.865 and 0.450 in this study, respectively (Sathianathen et al., 2019) (Table 2). These utility values were used to estimate the health outcomes and cost-effectiveness of the different treatment regimens.

### 2.5 Sensitivity analysis

To assess the robustness of the model results, deterministic sensitivity analyses (DSAs) were conducted by varying one model input or assumption at a time. The parameters were independently varied within a plausible range determined by either published data or by 95% confidence intervals. If not applicable, the values were varied by ± 20% of the corresponding base case value. Probabilistic sensitivity analysis (PSAs) was conducted to estimate the probability for different mHSPC treatment regimens to be cost-effective compared to ADT alone based on different WTP thresholds. A Monte-Carlo simulation with 5,000 iterations was conducted. Utility scores and cost were assumed to follow a beta and gamma distribution, respectively. The results were presented in a cost-effectiveness scatter plot and a cost-effectiveness acceptability curve (CEAC) comparing ADT with each comparator (Supplementary Figure S2).

## 3 Results

### 3.1 Base-case results

This study comparatively analyzed the cost-effectiveness of eight first-line treatment regimens for mHSPC (Table 3). The estimated 10-year total costs ranged from US$120,844 (ADT alone) to US$216,294 (darolutamide plus ADT with docetaxel), while the estimated 10-year QALYs were highest for enzalutamide plus ADT (4.55) and the lowest for ADT alone (3.01). The three regimens, ADT alone, abiraterone plus ADT and enzalutamide plus ADT formed the cost-effectiveness frontier in China, which indicates that these mHSPC treatment regimens might be cost-effective (Supplementary Figure S3). Serving as the control group, ADT monotherapy showed the lowest total cost (US$120,844) but the lowest quality-adjusted life-years (3.01 QALYs). Although enzalutamide combined with ADT yielded the highest QALYs (4.55), its ICER of US$44,107 per QALY slightly exceeded China’s WTP threshold with compromised cost-effectiveness in clinical practice. Interestingly, abiraterone plus ADT achieved the optimal balance between clinical benefits and costs with an ICER of US$17,437 per QALY. This ICER is significantly lower than China’s commonly used WTP thresholds of US$38,024 per QALY (three-time GDP *per capita*), making the regimen of abiraterone plus ADT the most cost-effective treatment for mHSPC. Moreover, the ICERs of rezvilutamide combined with ADT, abiraterone combined with docetaxel and ADT, apalutamide combined with ADT, darolutamide combined with docetaxel and ADT, docetaxel combined with ADT were US$49806/QALY, US$60,840/QALY, US$66,237/QALY, US$80,253/QALY and US$133,723/QALY in China, respectively, significantly exceeding China’s WTP threshold.

**TABLE 3:** Results of base case analysis.

Regimens	Cost, US$	Incr Cost	QALY	Incr QALY	C/E	ICER (US$/QALY)
ADT	120,844.68		3.01		40,191.70	
Abiraterone + ADT	140,344.01	19,499.33	4.12	1.12	34,023.03	17,437.16
Enzalutamide + ADT	188,920.78	68,076.10	4.55	1.54	41,519.87	44,107.26
Rezvilutamide + ADT	176,875.14	56,030.46	4.13	1.12	42,809.68	49,806.86
Abiraterone + docetaxel + ADT	177,013.57	56,168.88	3.93	0.92	45,042.50	60,840.48
Apalutamide + ADT	154,590.57	33,745.89	3.52	0.51	43,965.53	66,237.38
Darotamide + Docetaxel + ADT	216,294.74	95,450.06	4.20	1.19	51,546.99	80,253.15
Docetaxel + ADT	140,547.48	19,702.80	3.15	0.15	44,560.99	133,723.18

Abbreviations: ADT, androgen deprivation therapy; Incr, incremental; QALYs, quality adjusted life years; ICER, incremental cost-effectiveness ratio.

### 3.2 Sensitivity analyses results

In general, one-way sensitivity analyses found that drug costs of ARPIs and docetaxel, utility of PFS had considerable impacts on the ICERs of nearly all treatment regimens for mHSPC (Supplementary Figure S4; Supplementary Table S9). The other parameters included in the sensitivity analyses, such as the costs of ADRs, had minimal effect on the ICERs (Supplementary Figure S4). In particular, for docetaxel plus ADT, cost of docetaxel and cost of follow up per cycle were the most influential parameter on the ICER. The ICER of docetaxel plus ADT treatment on the basis of lower and upper values of each parameter were all higher than WTP threshold, in line with the base-case results. For abiraterone plus ADT, the parameter with the highest impact on the ICER was cost of abiraterone. When the cost of abiraterone is lower than US$5.601 per tablet, its ICER remains below the WTP threshold (three-time GDP *per capita* per QALY). Conversely, when above US$5.601/tablet, the ICER exceeds the WTP threshold.

**FIGURE 3:**
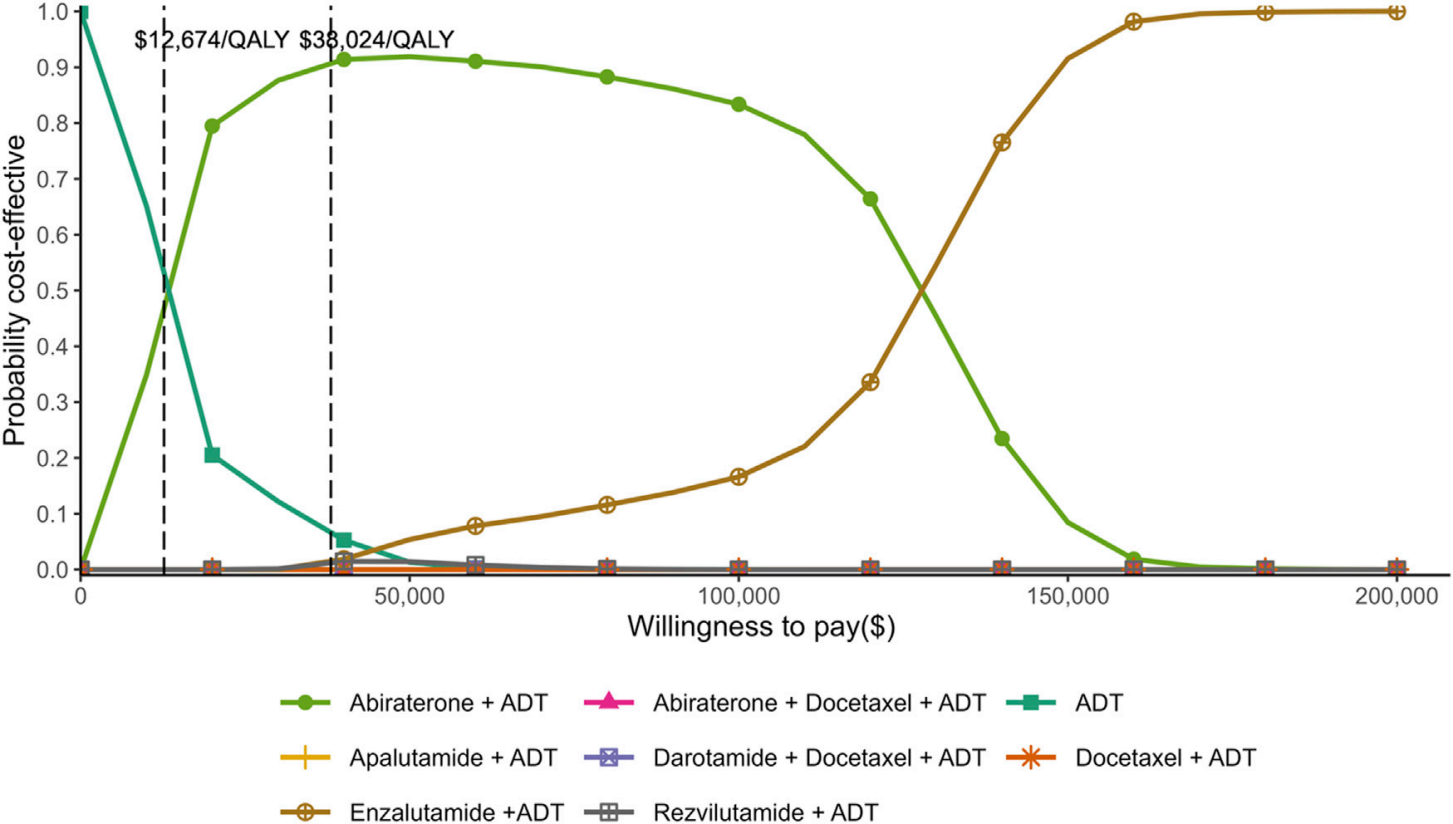
Cost-effectiveness acceptability curve illustrating the probability that each first-line treatment represents the most cost-effective strategy at varying willingness-to-pay thresholds.

**FIGURE 4:**
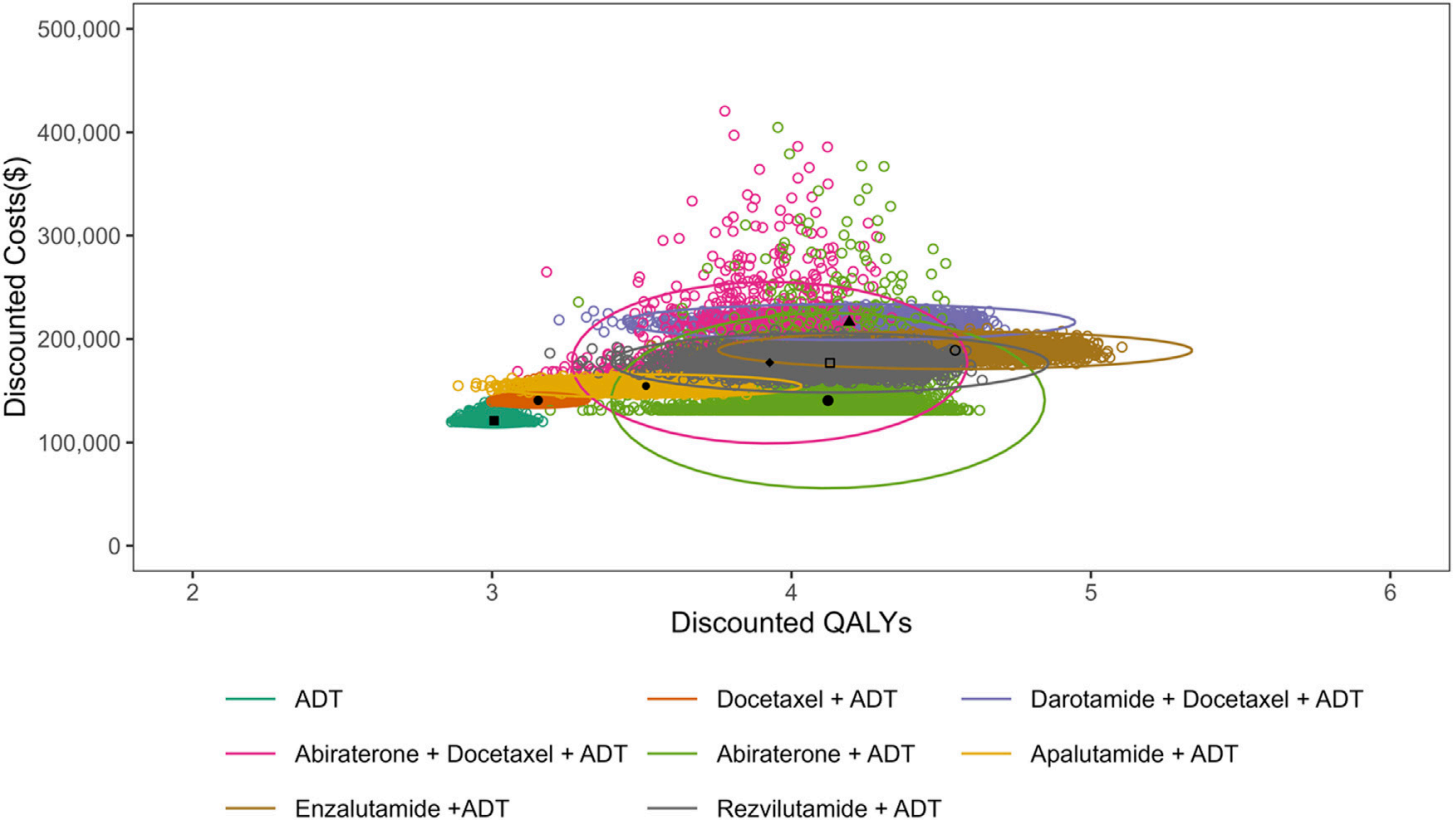
Probabilistic scatter plot of costs and QALYs for a cohort of 5,000 simulations. Ellipses represent the 95% confidence regions of the estimates.

The results of probabilistic sensitivity analysis showed that the probabilities of each treatment being cost-effective at different WTP thresholds (Figure 3). At a WTP threshold equivalent to US$12,674 (one-time GDP *per capita*) per QALY, ADT alone demonstrated the highest probability of being cost-effective, slightly exceeding the abiraterone plus ADT. At a threshold of US$38,024 (three-time GDP *per capita*) per QALY, abiraterone plus ADT exhibited the highest probability among the eight treatment regimens, significantly exceeding those of ADT alone and other treatment strategies. However, if WTP threshold were to substantially exceed three-time GDP *per capita* (e.g., US$150,000), enzalutamide plus ADT would emerge as the most cost-effective option. The distributions of costs and QALYs for each treatment regimen are plotted in Figure 4, providing a visual explanation for the CEAC results (Figure 3). Specifically, the cluster of points for ADT alone near the origin explains its value at low WTP. The north-east distribution of abiraterone plus ADT reflects a favorable cost-effectiveness trade-off at intermediate thresholds. Conversely, enzalutamide plus ADT, with the rightmost position, required very high WTP thresholds to become the optimal choice, given its highest cost and benefit profile.

## 4 Discussion

ADT remains a cornerstone treatment for mHSPC. Despite its high initial treatment response rates, ADT alone often fails to sustain disease control or achieve long-term survival, with most patients progressing to the mCRPC stage (Saad et al., 2024). To address this issue, combined regimens of ADT with ARPIs and/or docetaxel have been introduced into the clinic in recent years. Intriguingly, these doublet or triplet therapies have shown better survival outcomes than ADT alone, yet are accompanied by substantially higher costs and increased toxicity, making it hard for clinicians to choose the treatment regimen for mHSPC patients (Dzimitrowicz and Armstrong, 2022; Hoeh et al., 2023). It is therefore imperative to conduct robust cost-effectiveness studies for mHSPC treatment regimens to identify the optimal pathway that maximizes both survival and quality of life (Ávila et al., 2025).

This study represents the first comprehensive cost-effectiveness analysis of first-line treatment regimens for mHSPC from the perspective of the Chinese healthcare system. These mHSPC treatment regimens, including all novel antiandrogen drugs approved in China, are widely recommended as the first-line therapies in the Chinese Society of Clinical Oncology (CSCO) guideline. At a WTP threshold of one-time GDP *per capita*, ADT alone is likely the most cost-effective option, whereas the probability of being cost-effective was optimal for abiraterone plus ADT when the WTP was set at three-time GDP *per capita*. This shift is driven by two factors: (1) a sharp decline in abiraterone’s price following generic entry. Specifically, a $1 reduction in the price per tablet resulted in a decrease of $5,174 in the ICER; and (2) significantly lower rates of subsequent second-line therapy (approximately 30%) in patients receiving abiraterone plus ADT compared to those on docetaxel plus ADT or enzalutamide plus ADT, thereby reducing downstream expenditures for progressive disease. Notably, healthcare policies like China’s National Reimbursement Drug List (NRDL) negotiations have substantially improved abiraterone’s affordability, increased its clinical accessibility and utilization rates and subsequently enhanced its cost-effectiveness. These findings indicate that implementation of similar price-regulation mechanisms for novel antiandrogens (e.g., enzalutamide, rezvilutamide) or the promotion of domestic alternatives would improve their cost-effectiveness profiles.

Under healthcare resource constraints, clinicians must weigh therapeutic benefits against costs to optimize treatment selection. Consistent with cost-effectiveness literature, ARPI plus ADT regimens conferred greater QALYs than ADT alone, with enzalutamide plus ADT delivering the highest gain (4.55 QALYs). Furthermore, both abiraterone plus ADT with docetaxel and darolutamide plus ADT with docetaxel triplet regimens yielded superior QALYs compared to docetaxel plus ADT dual therapy. Unexpectedly, we observed that QALYs were lower with abiraterone plus ADT and docetaxel (3.93) versus abiraterone plus ADT (4.12), possibly due to docetaxel’s toxicity diminishing quality-of-life benefits and trial imbalances in high-burden patient enrollment.

In cost-effectiveness terms, abiraterone plus ADT demonstrated an ICER of US$17,437/QALY versus ADT monotherapy—below three-time China’s GDP *per capita* threshold (US$38,024). However, while enzalutamide plus ADT and rezvilutamide plus ADT increased QALYs, their ICERs reflect prohibitively high incremental costs that may impose substantial economic burdens on patients and payers. Triplet regimen analysis further revealed darolutamide plus ADT with docetaxel generated the highest ICER among all evaluated regimens, substantially exceeding the WTP thresholds and rendering it economically unattractive. These findings indicate that while combination therapies enhance clinical outcomes, their cost-effectiveness is not guaranteed. This balance depends on two key factors: (1) Incremental cost-utility ratios, exemplified by rezvilutamide’s substantial PFS/OS improvements counteracted by its launch price premium, highlight critical cost-effectiveness tradeoffs; (2) Toxicity-driven cost/QALY tradeoffs, particularly for docetaxel-containing regimens where adverse event management increases costs and toxicity disminishes QALY gains.

Our findings align with prior mHSPC cost-benefit studies from other regions. A 2021 cost-effectiveness analysis (Sung et al., 2021) demonstrated that given a WTP threshold of US$100,000/QALY and Veterans Affairs Federal Supply Schedule (VA-FSS) costing, abiraterone plus ADT delivered high-value care. However, this earlier report has some disadvantages over our research. The therapeutic paradigm for mHSPC has shifted toward triplet regimens, but this previous report did not include triplet regimens. Moreover, a critical divergence exists between our study and the 2021 research regarding the proportion of patients receiving second-line therapy-a key cost parameter. While our analysis derived this proportion directly from RCT evidence, the earlier study relied on modeled assumptions. Similarly, a US-based mHSPC cost-effectiveness analysis (Yoo et al., 2023), which did not include abiraterone plus ADT with docetaxel and rezvilutamide regimens, reported that abiraterone plus ADT was most cost-effective (ICER: US$21,247/QALY) at WTP threshold of US$100,000/QALY using VA-FSS costs. Furthermore, QALYs for all pharmacotherapeutic regimens in our study were consistently lower than those in prior studies. Specifically, ADT alone and apalutamide plus ADT yielded 3.01 and 3.52 QALYs, respectively. These discrepancies arise from methodological distinctions in survival data extraction. Earlier studies extrapolated PFS/OS curves using network meta-analysis hazard ratios, whereas to preserve granular trial details, including temporal survival dynamics and censoring event distributions, we reconstructed individual patient data via standard statistical methodology described by Guyot (Guyot et al., 2012). Interestingly, despite significant differences in WTP between China and the United States, the abiraterone plus ADT regimen demonstrates consistent cost-effectiveness advantages in pharmacoeconomic evaluations (Sung et al., 2021; Yoo et al., 2023). Specifically, the regimen achieved favorable ICER within both healthcare systems. This finding underscores the robustness of abiraterone plus ADT’s clinical value, as it maintains the highest probability of being cost-effective across a broad spectrum of WTP thresholds. Moreover, while there is a scarcity of CEAs for mHSPC in China, a 2019 publication is of particular relevance (Liu et al., 2019). That study demonstrated that docetaxel-based combination therapy was more cost-effective than abiraterone plus ADT at WTP threshold of three times China’s GDP *per capita*. Intriguingly, this conclusion reversed when the threshold was raised to eight times the GDP *per capita*, at which point abiraterone plus ADT became the more cost-effective option. This divergence from our own findings may be largely explained by a radical shift in drug pricing. Specifically, following its inclusion in China’s national drug price negotiation in 2016, the price of abiraterone has plummeted by 98.4%.

Our analysis has several limitations. First, the data in this study are obtained from several RCTs and network meta-analyses. The inclusion/exclusion criteria are not entirely consistent across different RCTs, which may introduce selection bias. The model was developed on reconstructed data without validation in the real-world settings, therefore limiting real-world applicability. Second, drug costs remain subject to healthcare policy fluctuations, e.g., price negotiations and generic entry, which could reduce incremental costs per QALY and enhance cost-effectiveness potential. Third, compared to docetaxel plus ADT or abiraterone plus ADT, follow-up durations for other regimens were comparatively shorter. Extended observation periods might alter survival and toxicity profiles. Fourth, our study did not included productivity loss-related indirect costs, which may underestimate the economic impact in working populations. Nevertheless, clinical and healthcare decision-makers primarily focus on direct medical expenditures, as these directly affect prescribing behaviors and healthcare funding sustainability.

## 5 Conclusion

The cost-effectiveness of eight first-line treatments for mHSPC was evaluated from the perspective of the Chinese healthcare system. This study demonstrated that abiraterone plus ADT may offer a clear cost-effectiveness advantage at a WTP threshold of three-time China’s GDP *per capita*, potentially positioning it as the optimal treatment for mHSPC. These findings could assist clinical decision-makers in striking a balance between improving health outcomes and controlling medical expenditures, particularly within the context of limited healthcare resources and funding.

## Data Availability

The raw data supporting the conclusions of this article will be made available by the authors, without undue reservation.
