# Supplementary material for: Cost-effectiveness analysis of eight first-line treatments for metastatic hormone-sensitive prostate cancer in China
Source: Front Pharmacol. 2025 Nov 28;16:1684966. doi: 10.3389/fphar.2025.1684966 (PMC12698425; doi:10.3389/fphar.2025.1684966)
Supplement: Supplementary file 1 [file Supplementaryfile1.docx]

**Supplementary Materials**

**Supplemental Figures and Tables**

**Figure S1. Progression-free survival (PFS) and overall survival (OS) curves for eight first-line treatment regimens for mHSPC in the base-case analysis of the partitioned survival model.** The smoothed curves represent model-predicted survival estimates based on the best-fitting distributions. The solid, dashed, and dotted lines represent the mean estimates, upper bounds, and lower bounds of the 95% confidence intervals, respectively.

**

**

**Figure S2. Scatter diagrams in the partitioned survival model.** Gamma distributions were used for costs, and beta distributions were used for utilities.





**Figure S3. Cost-effective frontier of the eight first-line treatment regimens for mHSPC.**





**Figure S4.** **Deterministic sensitivity analysis results of eight first-line treatment regimens for mHSPC.**





**Tables**

**Table S1. Health state transition probability.** The detailed information was included in an Excel table.

**Table S2. Characteristics of phase III clinical trials for mHSPC.**

| **Trial ID** | **Experimental** | **Comparator** | **Overall Population** | |
| --- | --- | --- | --- | --- |
|  |  |  | rPFS, HR (95%Cl) | OS, HR (95%Cl) |
| **GETUG-AFU 15** | Docetaxel+ADT | ADT | 0.69(0.55-0.87) | 0.88(0.68-1.14) |
| **CHAARTED** | Docetaxel+ADT | ADT | 0.62(0.51-0.75) | 0.72(0.59-0.89) |
| **STAMPEDE arm**  **(B, C, E)** | Docetaxel+ADT | ADT | 0.69(0.59-0.81) | 0.81(0.69-0.95) |
| **STAMPEDE arm G** | Abiraterone+ADT | ADT | 0.45(0.37-0.54) | 0.61(0.49-0.79) |
| **ENZAMET** | Enzalutamide+ADT | SNA +ADT | 0.40(0.33-0.49) | 0.67(0.52-0.86) |
|  | Enzalutamide+ Docetaxel+ADT | SNA+Docetaxel+ADT | 0.48(0.37-0.62) | 0.9(0.62-1.31) |
| **LATITUDE** | Abiraterone+ADT | ADT | 0·47(0·39–0·55) | 0·66(0·56-0·78) |
| **ARCHES** | Enzalutamide+ADT | ADT | 0.39(0.30-0.50) | 0.66 (0.53-0.81) |
|  | Enzalutamide+  Docetaxel+ADT | Docetaxel+ADT | 0.52(0.30-0.89) | 0.74(0.46-1.20) |
| **TITAN** | Apalutamide+ADT | ADT | 0.49(0.40–0.61) | 0.65(0.53-0.79) |
|  | Apalutamide+  Docetaxel+ADT | Docetaxel+ADT | 0.47(0.22-1.01） | 1.12(0.59-2.12） |
| **PEACE-1** | Abiraterone+  RT (+/-) + ADT | RT (+/-) +ADT | 0.54(0.46-0.64) | 0·82(0·69-0·98) |
|  | Abiraterone+Docetaxel+RT (+/-) +ADT | Docetaxel+RT (+/-) +ADT | 0.50(0. 39-0.61) | 0·75(0·59-0·95) |
| **CHART** | Rezvilutamide+ADT | SNA+ADT | 0.44(0.33-0.58) | 0.58(0.44-0.77) |
| **STAMPEDE arm** | Abiraterone+ADT | Docetaxel+ADT | 0.65(0.48–0.88) | 1.16(0.82-1.65) |
| **ARASENS** | Darolutamide+  Docetaxel+ADT | Docetaxel+ADT | NA | 0.68(0.57-0.80) |

Abbreviations: RT, radiotherapy; SNA, standard nonsteroidal antiandrogen (bicalutamide, nilutamide or flutamide); NA, not available.

**Table S3.** CHEERS 2022 Checklist. The Consolidated Health Economic Evaluation Reporting Standards (CHEERS) Checklist items were included when reporting economic evaluations of health interventions. CHEERS: Good Reporting Practices webpage: https://don-husereau.shinyapps.io/CHEERS/.

| **Topic** | **No.** | **Item** | **Inclusion status** |
| --- | --- | --- | --- |
| **Title** |  |  |  |
|  | 1 | Identify the study as an economic evaluation and specify the interventions being compared. | Yes |
| **Abstract** |  |  |  |
|  | 2 | Provide a structured summary that highlights context, key methods, results, and alternative analyses. | Yes |
| **Introduction** |  |  |  |
| **Background and objectives** | 3 | Give the context for the study, the study question, and its practical relevance for decision making in policy or practice. | Yes |
| **Methods** |  |  |  |
| **Health economic analysis plan** | 4 | Indicate whether a health economic analysis plan was developed and where available. | Yes/Model Overview |
| **Study population** | 5 | Describe characteristics of the study population (such as age range, demographics, socioeconomic, or clinical characteristics). | Yes/Model Overview |
| **Setting and location** | 6 | Provide relevant contextual information that may influence findings. | Yes/Model Overview |
| **Comparators** | 7 | Describe the interventions or strategies being compared and why chosen. | Yes/Model Overview |
| **Perspective** | 8 | State the perspective(s) adopted by the study and why chosen. | Yes/Model Overview |
| **Time horizon** | 9 | State the time horizon for the study and why appropriate. | Yes/Model Overview |
| **Discount rate** | 10 | Report the discount rate(s) and reason chosen. | Yes/Model Overview |
| **Selection of outcomes** | 11 | Describe what outcomes were used as the measure(s) of benefit(s) and harm(s). | Yes/Model Overview |
| **Measurement of outcomes** | 12 | Describe how outcomes used to capture benefit(s) and harm(s) were measured. | Yes/Model Overview |
| **Valuation of outcomes** | 13 | Describe the population and methods used to measure and value outcomes. | Yes/Model Overview |
| **Measurement and valuation of resources and costs** | 14 | Describe how costs were valued. | Yes/Medical Costs and health utilities |
| **Currency, price date, and conversion** | 15 | Report the dates of the estimated resource quantities and unit costs, plus the currency and year of conversion. | Yes/Medical Costs and health utilities |
| **Rationale and description of model** | 16 | If modelling is used, describe in detail and why used. Report if the model is publicly available and where it can be accessed. | Yes/Model Overview |
| **Analytics and assumptions** | 17 | Describe any methods for analysing or statistically transforming data, any extrapolation methods, and approaches for validating any model used. | Yes/Model Overview |
| **Characterising heterogeneity** | 18 | Describe any methods used for estimating how the results of the study vary for subgroups. | Yes/Sensitivity Analysis |
| **Characterising distributional effects** | 19 | Describe how impacts are distributed across different individuals or adjustments made to reflect priority populations. | Yes/Sensitivity Analysis |
| **Characterising uncertainty** | 20 | Describe methods to characterise any sources of uncertainty in the analysis. | Yes/Sensitivity Analysis |
| **Approach to engagement with patients and others affected by the study** | 21 | Describe any approaches to engage patients or service recipients, the general public, communities, or stakeholders (such as clinicians or payers) in the design of the study. | NA |
| **Results** |  |  |  |
| **Study parameters** | 22 | Report all analytic inputs (such as values, ranges, references) including uncertainty or distributional assumptions. | Yes/Table1 and 2 |
| **Summary of main results** | 23 | Report the mean values for the main categories of costs and outcomes of interest and summarise them in the most appropriate overall measure. | Yes/Table3 |
| **Effect of uncertainty** | 24 | Describe how uncertainty about analytic judgments, inputs, or projections affect findings. Report the effect of choice of discount rate and time horizon, if applicable. | Yes/Sensitivity Analyses Results |
| **Effect of engagement with patients and others affected by the study** | 25 | Report on any difference patient/service recipient, general public, community, or stakeholder involvement made to the approach or findings of the study | NA |
| **Discussion** |  |  |  |
| **Study findings, limitations, generalisability, and current knowledge** | 26 | Report key findings, limitations, ethical or equity considerations not captured, and how these could affect patients, policy, or practice. | Yes/Discussion |
| **Other relevant information** |  |  |  |
| **Source of funding** | 27 | Describe how the study was funded and any role of the funder in the identification, design, conduct, and reporting of the analysis | Yes/Funding |
| **Conflicts of interest** | 28 | Report authors conflicts of interest according to journal or International Committee of Medical Journal Editors requirements. | Yes/Conflicts of interest |

**Table S4. Estimates of background mortality rate for each age are provided in the China life table.**

| Age (years) | Background | Age (years) | Background | Age (years) | Background |
| --- | --- | --- | --- | --- | --- |
| 0 | 0.00382 | 34 | 0.00094 | 68 | 0.01864 |
| 1 | 0.00111 | 35 | 0.00103 | 69 | 0.02191 |
| 2 | 0.00063 | 36 | 0.00106 | 70 | 0.02557 |
| 3 | 0.00045 | 37 | 0.00114 | 71 | 0.02673 |
| 4 | 0.00037 | 38 | 0.00121 | 72 | 0.03094 |
| 5 | 0.00033 | 39 | 0.00134 | 73 | 0.03359 |
| 6 | 0.00032 | 40 | 0.00151 | 74 | 0.03744 |
| 7 | 0.00028 | 41 | 0.00155 | 75 | 0.04151 |
| 8 | 0.00028 | 42 | 0.00182 | 76 | 0.04219 |
| 9 | 0.00028 | 43 | 0.00189 | 77 | 0.05097 |
| 10 | 0.0003 | 44 | 0.00207 | 78 | 0.0562 |
| 11 | 0.00029 | 45 | 0.00231 | 79 | 0.06212 |
| 12 | 0.0003 | 46 | 0.00236 | 80 | 0.07428 |
| 13 | 0.00029 | 47 | 0.00254 | 81 | 0.07791 |
| 14 | 0.0003 | 48 | 0.00311 | 82 | 0.08581 |
| 15 | 0.00034 | 49 | 0.00328 | 83 | 0.09352 |
| 16 | 0.00035 | 50 | 0.00364 | 84 | 0.10363 |
| 17 | 0.00039 | 51 | 0.00375 | 85 | 0.111 |
| 18 | 0.00041 | 52 | 0.00398 | 86 | 0.11888 |
| 19 | 0.00043 | 53 | 0.00441 | 87 | 0.13007 |
| 20 | 0.00047 | 54 | 0.00498 | 88 | 0.14418 |
| 21 | 0.00047 | 55 | 0.00518 | 89 | 0.15687 |
| 22 | 0.0005 | 56 | 0.00564 | 90 | 0.17652 |
| 23 | 0.00054 | 57 | 0.00609 | 91 | 0.18524 |
| 24 | 0.00056 | 58 | 0.00681 | 92 | 0.20211 |
| 25 | 0.00058 | 59 | 0.00767 | 93 | 0.20735 |
| 26 | 0.00057 | 60 | 0.00854 | 94 | 0.20891 |
| 27 | 0.00059 | 61 | 0.00938 | 95 | 0.22001 |
| 28 | 0.00061 | 62 | 0.01038 | 96 | 0.22099 |
| 29 | 0.00068 | 63 | 0.01112 | 97 | 0.20484 |
| 30 | 0.0007 | 64 | 0.01301 | 98 | 0.19813 |
| 31 | 0.00077 | 65 | 0.01421 | 99 | 0.25765 |
| 32 | 0.00081 | 66 | 0.01474 | 100 | 0.45435 |
| 33 | 0.00083 | 67 | 0.01723 |  |  |

**Table S5. Drug dose and costs of eight treatment strategies.**

| Drug name | Dose | Strength | Route | Price |
| --- | --- | --- | --- | --- |
| Abiraterone | 1000mg/d | 250mg | Oral | $ 87.64-$1827.98 |
| Enzalutamide | 160mg/d | 40mg | Oral | $ 189.61-$ 1094.83 |
| Darotamide | 1200mg/d | 300mg | Oral | $ 830.56 |
| Rezvilutamide | 240mg/d | 80mg | Oral | $ 831.74 |
| Apalutamide | 240mg/d | 60mg | Oral | $ 815.73 |
| Docetaxel | 75mg/m^2^×1.72m^2^ every 3 weeks | 1ml:20mg | IV | $ 3.17-$ 99.32 |
| Prednisone | 10mg/d | 5mg | Oral | $ 0.59-$ 5.06 |
| Dexamethasone | 16mg/d | 0.75mg | Oral | $ 0.97-$ 1.52 |
| Goserelin | 3.6 mg per month | 10.8mg | SC | $ 349.69 |
| Leuprorelin | 3.75 mg per month | 3.75mg | IM | $ 126.95-$ 223.77 |
| Triptorelin | 5 mg per month | 15mg | SC | $ 405.25 |

Abbreviations: IV, intravenous injection; SC, subcutaneous injection; IM, intramuscular injection

**Table S6. Selected Subsequent Therapy for Prostate Cancer.**

| **First-line treatment strategies** | **Proportion of second-line treatments** | **Second-line treatment strategies** | |
| --- | --- | --- | --- |
| **Abiraterone+** **Docetaxel+ADT** | 74% | Abiraterone+ADT | 16% |
|  |  | Enzalutamide+ADT | 40% |
|  |  | Docetaxel+ADT | 40% |
| **Abiraterone+ADT** | 30% | Abiraterone+ADT | 3% |
|  |  | Enzalutamide+ADT | 10% |
|  |  | Docetaxel+ADT | 24% |
| **Enzalutamide+ADT** | 67.10% | Abiraterone+ADT | 27.50% |
|  |  | Enzalutamide+ADT | 0% |
|  |  | Docetaxel+ADT | 26.90% |
|  |  | Enzalutamide+ADT | 2% |
|  |  | Docetaxel+ADT | 14% |
| **Darotamide+** **Docetaxel+ADT** | 56.80% | Abiraterone+ADT | 35.60% |
|  |  | Enzalutamide+ADT | 15.20% |
|  |  | Docetaxel+ADT | 32.70% |
| **Rezvilutamide+ADT** | 27% | Abiraterone+ADT | 17% |
|  |  | Enzalutamide+ADT | 2% |
|  |  | Docetaxel+ADT | 14% |
| **Apalutamide+ADT** | 47% | Abiraterone+ADT | 14.50% |
|  |  | Enzalutamide+ADT | 6.50% |
|  |  | Docetaxel+ADT | 26.80% |
| **Docetaxel+ADT** | 59% | Abiraterone+ADT | 26.40% |
|  |  | Enzalutamide+ADT | 20.10% |
|  |  | Docetaxel+ADT | 13.60% |
| **ADT** | 73% | Abiraterone+ADT | 1% |
|  |  | Enzalutamide+ADT | 23% |
|  |  | Docetaxel+ADT | 34.80% |

**Table S7. Laboratory tests and imaging cost.**

| **Laboratory tests and imaging** | **Cost** | **Frequency** | **Details** | **Source** |
| --- | --- | --- | --- | --- |
| Serum PSA | $ 9.13 | Every 3 months | Total PSA (T-PSA), free PSA (F-PSA) and free/total PSA (F/T-PSA) | JIANGSU Provincial Health Commission |
| Serum testosterone | $ 9.13 | Every 3 months | Testosterone | JIANGSU Provincial Health Commission |
| Blood routine | $ 2.53 | Every 3 months | Red blood cell count, white blood cells count, lymphocyte count, hemoglobin count, and platelets | JIANGSU Provincial Health Commission |
| Blood biochemistry | $ 31.04 | Every 3 months | ALT, AST, GGT, total  bilirubin, direct bilirubin, AKP, blood urea nitrogen, total protein, albumin, creatine, blood sugar, lactate dehydrogenase, K^+^, Na^+^, Ca^2＋^, Mg^2＋^, Cl^-^ | JIANGSU Provincial Health Commission |
| Coagulation parameters | $ 18.54 | Every 3 months | APTT, PT, FIB, TT, INR | JIANGSU Provincial Health Commission |
| Urinalysis | $ 3.72 | Every 3 months | White blood cells, red blood cells, urine protein | JIANGSU Provincial Health Commission |
| Bone scans | $ 44.94 | Annually | Bone scans | JIANGSU Provincial Health Commission |
| Contrast-enhanced/CT MRI | $ 192.48 | Annually | Contrast-enhanced CT/ MRI for chest and pelvic | JIANGSU Provincial Health Commission |
| PET-CT | $ 881.49 | Annually | PET-CT | JIANGSU Provincial Health Commission |

**Table S8. Grade 3/4 adverse reaction data and theapy for first-line systemic therapy in mHSPC.**

|  | **Darotamide+Docetaxel+ADT** | **Abiraterone+Docetaxel+ADT** | **Abiraterone+ADT** | **Apalutamide+ADT** | **Enzalutamide+ADT** | **Rezvilutamide+ADT** | **Docetaxel+ADT** | **ADT** | **Duration** | **Drugs used** | **Cost for AE per duration** |
| --- | --- | --- | --- | --- | --- | --- | --- | --- | --- | --- | --- |
| **Grade≥3 adverse event** | 44.8% | 63% | 63% | 53% | 42% | 51.4% | 68% | 34% | - | - | - |
| **Fatigue** | 33% | 3.0% | 2.0% | 1.5% | 6.0% | 0.0% | 33% | 2.0% | 7 d | Buzhong Yiqi Pill/10 pills po tid | $ 15.86 |
| **Back pain** | NA | NA | 3.0% | 2.3% | NA | 0.3% | NA | 3.0% |  | Flurbiprofen gel patch/ 1 patch po bid | $ 107.17 |
| **Asthenia** | NA | NA | 1.0% | 1.9% | NA | NA | NA | 1.0% |  | Buzhong Yiqi Pill/ 10 pills po tid | $ 15.86 |
| **Febrile neutropenia** | 7.8% | 5.0% | NA | NA | 7.0% | NA | 5.0% | NA | 14 d | Piperacillin and tazobactam/ 4.5 g ivgtt q8h and Human granulocyte stimulating factor/ 150 μg ih qd | $ 1497.88 |
| **Neutropenia** | 33.7% | 10.0% | 1.0% | NA | 6.0% | NA | 34.2% | 1.0% | 5 d | Human granulocyte stimulating factor/ 150 μg ih qd | $ 1456.6 |
| **Bone pain** | NA | NA | 4.0% | 1.1% | 0.2% | 1.9% | NA | 3.0% |  | Celecoxib/ 200 mg po bid | $ 61.46 |
| **Hypertension** | 6.4% | 22.0% | 21.0% | 8.4% | 8.0% | 3.0% | 13.0% | 10.0% | 8 w | Benazepril/ 10 mg po qd | $ 332.00 |
| **Hypokalemia** | NA | NA | 12.0% | NA | NA | 3.4% | NA | 1.0% | 4 w | Potassium chloride/ 1 g po bid | $ 17.8 |
| **Anemia** | 4.8% | NA | 3.0% | 1.7% | 1.0% | 3.7% | 5.1% | 4.0% |  | Ferrous Succinate/ 0.2g po qd and Folic Acid / 5mg po qd | $ 75.54 |
| **ALT increased** | 2.8% | NA | 5.0% | NA | NA | 2.2% | 1.7% | 1.0% |  | Compound Glycyrrhizin/ 3 tablets po tid | $ 409.5 |
| **AST increased** | 2.6% | NA | 4.0% | NA | NA | 2.2% | 1.1% | 1.0% |  |  |  |

Abbreviations: NA, not available.

**Table S9. Detailed information of the deterministic sensitivity analysis.** This was included in an Excel table.
